# Integrated point-of-care testing (POCT) of HIV, syphilis, malaria and anaemia in antenatal clinics in western Kenya: A longitudinal implementation study

**DOI:** 10.1371/journal.pone.0198784

**Published:** 2018-07-20

**Authors:** Nicole Young, Miriam Taegtmeyer, George Aol, Godfrey M. Bigogo, Penelope A. Phillips-Howard, Jenny Hill, Kayla F. Laserson, Feiko Ter Kuile, Meghna Desai

**Affiliations:** 1 Department of International Public Health, Liverpool School of Tropical Medicine, Liverpool, United Kingdom; 2 Department of Clinical Sciences, Liverpool School of Tropical Medicine, Liverpool, United Kingdom; 3 Kenya Medical Research Institute, Center for Global Health Research, Kisumu, Kenya; 4 Division of Global Health Protection, Center for Global Health, Centers for Disease Control and Prevention, Atlanta, GA, United States of America; 5 Malaria Branch, Division of Parasitic Diseases and Malaria, Center for Global Health, Centers for Disease Control and Prevention, Atlanta, GA, United States of America; University of Toronto Dalla Lana School of Public Health, CANADA

## Abstract

**Background:**

In sub-Saharan Africa, HIV, syphilis, malaria and anaemia are leading preventable causes of adverse pregnancy outcomes. In Kenya, policy states women should be tested for all four conditions (malaria only if febrile) at first antenatal care (ANC) visit. In practice, while HIV screening is conducted, coverage of screening for the others is suboptimal and early pregnancy management of illnesses is compromised. This is particularly evident at rural dispensaries that lack laboratories and have parallel programmes for HIV, reproductive health and malaria, resulting in fractured and inadequate care for women.

**Methods:**

A longitudinal eight-month implementation study integrating point-of-care diagnostic tests for the four conditions into routine ANC was conducted in seven purposively selected dispensaries in western Kenya. Testing proficiency of healthcare workers was observed at initial training and at three monthly intervals thereafter. Adoption of testing was compared using ANC register data 8.5 months before and eight months during the intervention. Fidelity to clinical management guidelines was determined by client exit interviews with success defined as ≥90% adherence.

**Findings:**

For first ANC visits at baseline (n = 529), testing rates were unavailable for malaria, low for syphilis (4.3%) and anaemia (27.8%), and near universal for HIV (99%). During intervention, over 95% of first attendees (n = 586) completed four tests and of those tested positive, 70.6% received penicillin or erythromycin for syphilis, 65.5% and 48.3% received cotrimoxazole and antiretrovirals respectively for HIV, and 76.4% received artemether/lumefantrine, quinine or dihydroartemisinin–piperaquine correctly for malaria. Iron and folic supplements were given to nearly 90% of women but often at incorrect doses.

**Conclusions:**

Integrating point-of-care testing into ANC at dispensaries with established HIV testing programmes resulted in a significant increase in testing rates, without disturbing HIV testing rates. While more cases were detected and treated, treatment fidelity still requires strengthening and an integrated monitoring and evaluation system needs to be established.

## Background

In sub-Saharan Africa (SSA), the most preventable leading causes of maternal mortality and adverse pregnancy outcomes are maternal HIV, syphilis, malaria and anaemia [1–4], with co-infections common [5–9]. SSA has an estimated maternal HIV prevalence of 5.3%, representing 92% of the world’s HIV positive pregnant women [10, 11]. Roughly 24% of deaths in women during pregnancy or post-partum are attributable to untreated HIV [1]. Maternal syphilis in the African region has a prevalence of 1.7%, which constitutes 63.1% of infections in pregnancy globally [2]. Syphilis is associated with spontaneous miscarriage, stillbirth, preterm birth, low birthweight, neonatal death, and congenital infection in infants [2]. The risk for both HIV and syphilis mother-to-child transmission cumulates with duration of exposure in utero [12, 13] and late treatments of syphilis may not avert damage to foetal development [14]. Where malaria is endemic, 45% of pregnancies would be infected [3, 15]. Starting from early gestation throughout, malaria parasitaemia is associated with anaemia, intrauterine growth restriction, preterm delivery, foetal loss, neonatal and infant mortality [3, 16–19]. Anaemia is also most prevalent in Africa: about 46% of pregnant women are anaemic and 1.5% are severely anaemic [20]. Anaemia during pregnancy causes fatigue and is associated with increased risk of post-partum haemorrhage, maternal mortality, low birth weight, and perinatal mortality [21–23]. There is substantial evidence that iron deficiency early in pregnancy (first and second trimester) contributes to higher risk of pre-term delivery and lower birth weight than if it occurred later [23, 24]. Thus, early prevention, diagnosis and treatment is key to protect women and their pregnancies. Many of these conditions do not exist in isolation and having one disease or condition may also be a risk factor for another: malaria is more common in women with HIV [25–27]; risk of HIV transmission is increased through genital ulcer disease [5]; malaria is a major risk factor for anaemia [28]; approximately 26% of severe anaemia in pregnant women is attributable to malaria and can be reduced by 38% from preventing malaria infection in pregnancy [3]. Because of their individual and combined contribution to disease, addressing these conditions together, as early as possible during pregnancy is an essential goal of antenatal care (ANC) [29].

Since 2001, Kenya has adopted the World Health Organization’s (WHO) focused ANC guidelines which call for a minimum of four visits [30] (recently updated to eight ‘contacts’ by WHO [29]). For the first visit, Kenyan guidelines recommend screening for HIV, syphilis and anaemia, urinalysis, and blood typing (together known as the ANC profile). To prevent the consequences of malaria in pregnancy, the current strategy recommends parasitic diagnosis of malaria only if the patient is febrile, together with the use of insecticide-treated nets (ITNs) and ANC administered intermittent preventive therapy with sulfadoxine-pyrimethamine (IPTp-SP). Expanding malaria testing at the first ANC visit to all pregnant women, regardless of fever [31–33], may be an attractive strategy in parts of Africa for various reasons including concerns about the impact of high grade SP resistance [34] or that some populations may no longer require IPTp because of marked reductions in malaria transmission. Furthermore, IPTp alone may be an inadequate intervention because women who attend ANC in the first trimester are not eligible for SP, and the uptake of IPTp has been low, with only half of pregnant women receiving at least one of three or more recommended doses of SP [19, 35, 36].

Rates of testing for syphilis and anaemia remain low among pregnant women in Kenya [37–39] even though 95% of pregnant women attend at least one ANC visit [40]. This is largely because most women attend ANC at dispensaries (tier-2 health facilities in the periphery) which do not typically stock diagnostic tests (except for HIV) and are referred, with additional costs and time implications, to distant facilities with laboratory capacity to complete their ANC profile [41, 42]. The difference in availability between tests for HIV and those for syphilis and anaemia reflects the high international priority and substantial financial support given to vertical HIV programmes resulting in parallel procurement and supervisory systems developed to ensure their accessibility and use [43, 44]. Syphilis and haemoglobin screening, despite the strong evidence for their clinical effectiveness [45–47], lack such donor advocacy and prioritization and suffer inadequate coverage [43, 44].

Affordable and reliable rapid point-of-care tests (POCTs) that require minimal training and equipment are available to fulfil antenatal screening requirements. Their simplicity and immediacy of results greatly benefit resource-constrained settings by allowing same-day initiation of management of conditions and their co-infections. POCTs have been instrumental in the scale-up of prevention of mother-to-child-transmission (PMTCT) of HIV services, achieving over 90% of HIV testing coverage in women who attend ANC [40]. The skills healthcare workers have gained from HIV testing can be expanded to integrate the use of POCTs for syphilis, malaria and anaemia at dispensaries so coverage of diagnosis and timely treatment can be improved.

This longitudinal implementation study quantitatively evaluated the adoption and fidelity of a programme intervention integrating point-of-care testing (POCT) for HIV, syphilis, malaria and anaemia at ANCs in dispensaries in western Kenya. We evaluated whether integrated POCT can increase the proportion of pregnant women tested and treated correctly for each of the respective conditions at first ANC visit. Other implementation outcomes of appropriateness, acceptability and feasibility will be reported elsewhere.

## Methods

### Study setting

The study was conducted between December 2014 and August 2015 in the site of the KEMRI and CDC Health and Demographic Surveillance System (HDSS) in Siaya County, western Kenya. The population is 95% ethnically Luo, rural, and lives through subsistence farming and local trading [48]. At the time of the study, there were 37 public health facilities in the study area: one district hospital, nine health centres and 27 dispensaries. Dispensaries comprise the lowest level (tier-2) of the formal health system and offer basic maternal and child health services, rudimentary out-patient curative care and support care for HIV positive patients. Personnel typically include one to two nurses trained to certificate (at least 30 months training post-secondary) or diploma level (at least 36 months training post-secondary), a part-time clinical officer (36 months training post-secondary), an HIV testing counsellor and some support staff. The dispensaries receive approximately 40 antenatal visits per month.

### Health facility evaluations pre-intervention

Initial facility assessments were conducted to assess capacity to offer POCTs in nine health centres and 24 dispensaries within the HDSS area (three dispensaries were not assessed). Facility evaluation covered information such as facility infrastructure, client load, number and type of antenatal health workers, testing services and client flow. The assessment used a survey and observation checklist adapted from the Demographic and Health Surveys (DHS) Program [49]. Instruments were pre-tested outside the catchment area and were carried out by trained data collectors.

Seven dispensaries were then purposively selected for inclusion in the study based on the following criteria: absence of other ongoing studies with pregnant women, geographic spread within the visual map area of HDSS, the number of monthly antenatal visits identified through retrospective register review, and willingness of the facility to participate. All seven facilities’ ANC clinics routinely conducted HIV testing, two irregularly conducted anaemia testing, and three irregularly conducted malaria testing. Lack of test supplies was the main reason for not conducting ANC testing for syphilis and anaemia.

### Implementation of programme

Integration here is defined as provision of all four tests concurrently, by a single healthcare worker, during a woman’s first ANC visit. In facilities that have specialized HIV testing counsellors, the four tests may be done by the counsellor instead of the ANC nurse. All the facilities’ ANC healthcare workers were given a competency-based training either at a central location or on-site. All participants received training manuals and job aid testing placemats with step-by-step instructions (S1 Fig). Training included using one finger-prick blood draw to run all four tests per standard operating procedures, safety, and appropriate preventive care and clinical management of positive results following Kenyan guidelines as summarised in Table 1:

**Table 1:** Appropriate clinical management for positive test results and preventive care at first ANC visit [50].

Condition	For treatment	For prevention
HIV	Initiate PMTCT; counsel; give cotrimoxazole and start triple therapy with antiretrovirals	
Syphilis	Provide single dose of 2.4 MU benzathine penicillin or if penicillin allergic and unable to access penicillin desensitization, give erythromycin 500 mg three times daily for seven days and counsel on partner notification	
Malaria	Uncomplicated malaria: give quinine in first trimester; give artemether/lumefantrine (AL), quinine or dihydroartemisinin-piperaquine (DP) in second or third trimester; clinical severe malaria: refer to hospital	Give directly observed IPTp-SP for malaria for women in second or third trimester not on cotrimoxazole; give ITN and advise to sleep under it.
Anaemia	Mild anaemia (Hb <10 g/dL): give 120 mg daily elemental iron; moderate anaemia (Hb 5–7.9 g/dL): as above and provide additional iron dextran; severe anaemia (Hb is <5 g/dL): refer to hospital	Not anaemic (Hb >10 g/dL): give 65 mg daily elemental iron
Neural tube defects		Low dose (0.4 mg) folic acid daily; if low dose folic acid is not available, high dose (5 mg) tablets should not be administered with SP but can be taken 14 days following administration of IPTp-SP.

ANC: antenatal care; POCT; point-of-care testing; PMTCT: prevention of mother-to-child transmission; IPTp-SP: intermittent preventive therapy in pregnancy with sulfadoxine-pyrimethamine; ITN: insecticide-treated net

Study facilities used existing HIV drugs and HIV POCTs supplied by the Government of Kenya per its standard national algorithm at the time: HIV (1+2) Antibody Colloidal Gold (KHB, Shanghai Kehua Bio-engineering Co Ltd, China) for screening, First Response HIV-1-2 kits (Premier Medical Corporation Ltd., Kachigam, India) for confirmation and Uni-Gold™ (Trinity Biotech, Ireland) for tie-breaking. Iron, folic acid, SP and malaria treatment drugs were also supplied free of charge by the government. The study supplied the facilities with POCTs for syphilis (SD BIOLINE Syphilis 3.0 test for antibodies against *Treponema pallidum*, Standard Diagnostic Inc., Korea), malaria (CareStart™ Malaria HRP2 Pf, AccessBio, USA) and haemoglobin concentrations (HemoCue® Hb 201+, HemoCue AB, Sweden) and ensured no stock-outs. For each lot number, 1% of the tests were selected at random and validated at KEMRI/CDC’s HIV reference laboratory in Kisumu, western Kenya, using known positive and negative samples. HemoCue® machines were calibrated every three months. The study also provided Brannan^TM^ triple timers, gloves, and benzathine penicillin for treating syphilis based on projected prevalence of syphilis in the area.

### Data collection and outcome indicators

Photographs of routine ANC registers were taken for 8.5 months before and eight months during intervention and the data were double-entered into the study database. There was no distinct column for recording malaria results in the registers so no reliable data existed pre-intervention and a column was added to facilitate collection of malaria test results during the intervention. From the registers, testing uptake was assessed among women aged 15–49 years below 28 gestational weeks of pregnancy (<28gw) attending their first ANC visit. The gestational age was estimated using date of last menstrual period (LMP) or fundal height if LMP was unknown. The evaluation was done in this group of women because adverse pregnancy outcomes are most preventable with early treatment and so we concentrated on outcomes in this population.

During the intervention, these women were also asked to participate in exit interviews with trained research staff following their visits. Written informed consents were obtained. For illiterate women, the consent forms were read to the women in the presence of a literate witness and verbal consents with thumb prints and witness signatures were obtained. Women who refused to participate or were unable to provide informed consent because of mental or physical disability were excluded. Those who consented were interviewed for 20–30 mins in a private area at the facility. Interviews asked whether women were given information about the blood tests, test results, any preventive care or treatments (including detailed information on type and dosage of drugs not captured by ANC registers) using picture cards or observed drugs by interviewers, counselling, and advice for partner notification.

Quality assessments (QA) through observed proficiency testing of healthcare workers to correctly perform rapid tests per manufacturers’ instructions were done immediately after initial training and at three, six and nine months (shortly after eight months of the intervention) using a 57-step checklist (S1 Text) by trained research staff. Those who performed ≥90% of the checklist correctly received reminders for any missed steps and proceeded to implement integrated POCT. Those who did not reach ≥90% were re-trained until proficient.

Endpoints for assessing programme adoption and implementation fidelity are summarised in Table 2. Adoption is defined as “the action to try or employ an innovation…also may be referred to as ‘uptake’” and fidelity is defined as the “degree to which [the] intervention was implemented as intended by program developers and the quality of program delivery” [51]. Success was set at ≥90% in line with global testing and treatment targets for HIV and syphilis [52, 53]. Degrees of under-reaching the success target were categorised as follows: under-reached 60% to <90%, very under-reached 40% to <60% and severely under-reached <40%. Because introducing new services may affect existing services, synergy of integrating POCT with HIV testing was determined by change of HIV testing rates before and after the intervention.

**Table 2:** Indicators of adoption, fidelity, and proposed success endpoints [51].

Indicators of adoption and fidelity		Indicator measure	Data source	Success endpoints
Adoption	Testing uptake and synergy into HIV programme	% of pregnant women tested for syphilis, malaria and anaemia by POCTs; unchanged or improved % of women tested for HIV	ANC register	≥ 90% tested; ≥0% change in HIV testing rates
Fidelity	Clinical management	% of pregnant women who receive appropriate: a) preventive care b) correct management for test positive cases	Exit interviews	≥90% received
	Information giving	% of pregnant women who were given: a) information about the four tests b) test results c) HIV counselling d) syphilis partner notification advice	Exit interviews	≥90% given/counselled/advised
	Health worker proficiency of POCT	Proficiency scores measuring the ability of the health care worker to correctly perform rapid diagnostic tests per manufacturer guidelines	Proficiency scores (%)	≥90% on check-list

POCTs: point-of-care tests; POCT: point-of-care testing; ANC: antenatal care

### Data analysis

Quantitative data were entered in Microsoft Excel 2016. Descriptive statistics and analyses were done in Stata 14. The command *metan* was used to graphically display before and during testing proportions. For categorical variables, proportions were calculated and Chi^2^ was used to test for associations. For non-normal distributions of continuous data, medians and inter-quartile ranges (IQR) were calculated and Wilcoxon rank-sum tests were used to test for associations. Box and whisker plots of proficiency tests scores were created in Microsoft Excel 2016. Using individual and facility level variables collected from the study, we explored factors associated with women not having a complete testing profile. Relative risks of having an incomplete ANC profile were obtained for each variable by fitting a log-binomial generalized estimating equation model that took facility clustering into account. Variables that had a significance level of <0.20 in the univariate analysis were considered for inclusion. Multicollinearity of selected variables was tested by using the variance inflation factor (VIF) command in Stata. Variables with a tolerance value (1/VIF) <0.1 were considered collinear with one of the other independent variables.

### Ethical considerations

The protocol was reviewed and approved by the scientific and ethical steering committees of the Kenya Medical Research Institute (protocol 2271) and the Liverpool School of Tropical Medicine Ethics Committee (protocol 14.017). For U.S. CDC, while this activity was determined to be human subjects research, CDC staff involvement did not constitute engagement in human subjects’ research, thus not requiring human subjects research review by the CDC institutional review board.

## Results

### Increase in testing uptake (ANC register data)

**Fig 1:**
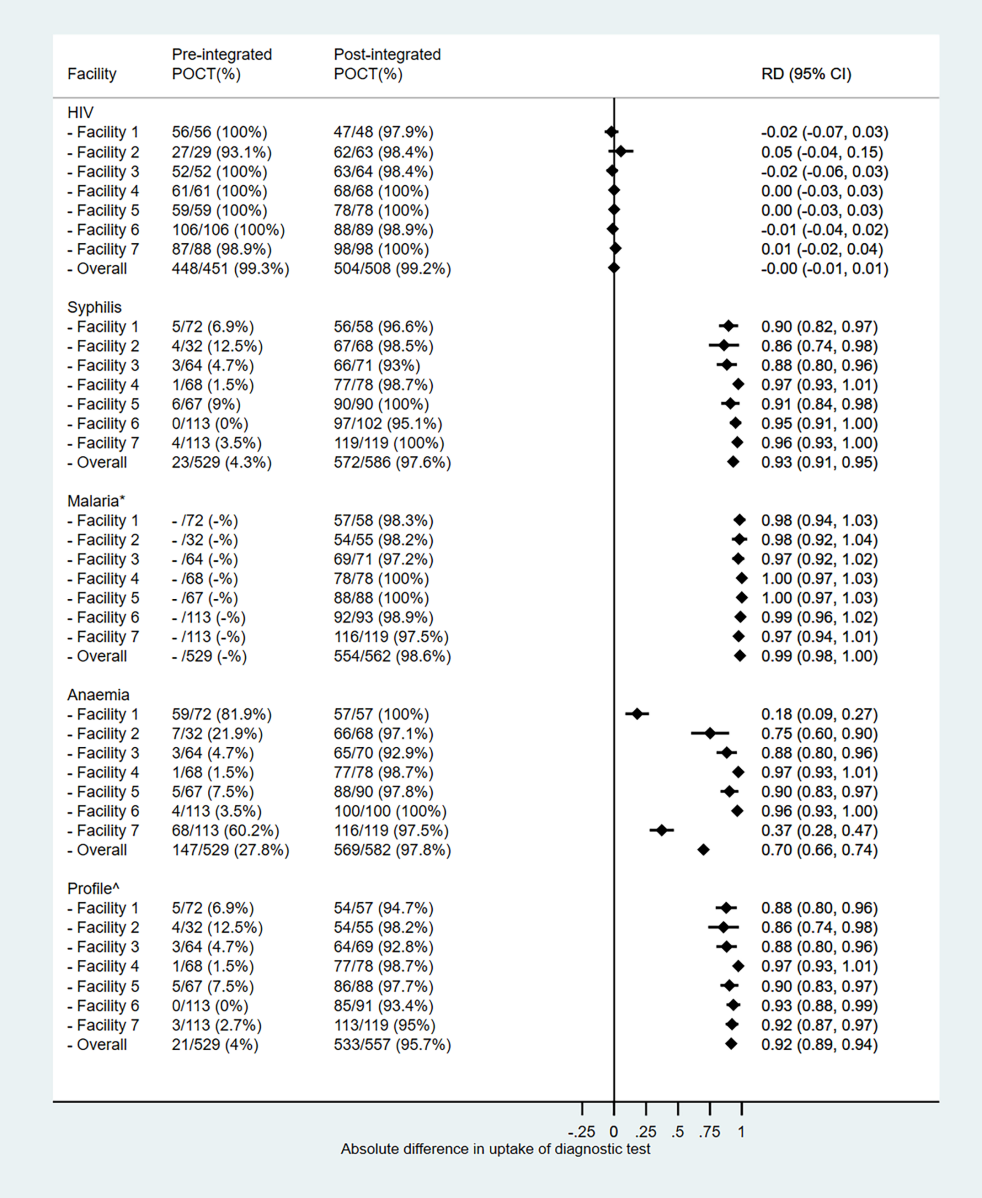
Proportion tested for condition pre (n = 529) and during (n = 586) integrated POCT programme by facility. *No data on number of women tested for malaria were available from the antenatal care registers before the intervention and therefore pre- and post-proportions were not comparable; ^profile pre-integrated POCT includes HIV, syphilis and anaemia; profile during integrated POCT includes HIV, syphilis, malaria and anaemia; POCT: point-of-care testing; RD: risk difference, interpreted as the difference in testing proportions before and during integrated POCT.

During 8.5 months before the integrated POCT programme, the seven facilities received 2279 ANC visits (median: 37, IQR: 28–37 visits per month) and 698 of them (median: 11, IQR: 8–11 visits per month) were first visits. Of the first visits, 529 (75.7%) women were <28gw and aged 15–49 years. During the eight months of integrated POCT programme, 2240 ANC visits were made at the seven facilities (median: 38, IQR: 32–38 visits per month). Of these, 728 were first ANC visits (median: 13, IQR: 10–13 visits per month), and 586 (80.5%) were by women <28gw aged 15–49. HIV testing rates remained over 90% in all facilities both before and during integrated POCT period. For syphilis and anaemia, overall testing proportions increased from 4.3% (mean: 5.4%, SD: 4.0%, range: 0–12.5%) and 27.8% (mean: 25.9%, SD: 29.8%, range: 1.5–81.9%) respectively to over 97%. The variations in syphilis and anaemia testing pre-intervention reflect the inconsistent availability of test supplies within and between facilities. Facility one and seven had noticeably higher testing rates for anaemia because they received test supplies from the district hospital and an external partner respectively. Malaria testing also reached over 97% in all facilities during the intervention (Fig 1).

#### New infections and co-infections picked up by POCTs

Among the 529 pregnancies evaluated pre-intervention, 31 new HIV infections, no syphilis infections, and 58 anaemic women (Hb <10 g/dL) were detected. There were 78 women with known HIV positive statuses.

Among the 586 pregnancies during integrated POCT, 41 new HIV infections, 18 syphilis infections, 177 malaria infections, and 223 anaemic women were detected. There were 77 women with known HIV positive statuses (Table 3). Among 118 HIV positive women, co-infections with syphilis were found in five (4.2%) and with malaria in 30 (25.4%). Among 223 anaemic women, malaria was detected in 90 (40.4%). One woman tested positive for syphilis, malaria and anaemia while none tested positive for all four conditions. Among HIV known positives, one woman was tested syphilis positive and anaemic.

**Table 3:** Test positivity rates and demographic characteristics of women aged between 15–49 years and <28 weeks pregnant, based on data from ANC registers 8.5 months before and 8 months during integrated POCT programme.

**Conditions**	**Before integrated** **POCT (n = 529)**	**During integrated POCT (n = 586)**			
	All	All	Not interviewed n = 106	Interviewed n = 480	P-value*
HIV	n = 529	n = 585	n = 105	n = 480	
known positive	78/529 (14.7%)	77/585 (13.2%)	16/105 (15.2%)	61/480 (12.7%)	0.487
tested for HIV	448/451 (99.3%)	504/508 (99.2%)	87/89 (97.8%)	417/419 (99.5)	0.087
tested positive	31/448 (6.9%)	41/504 (8.1%)	12/87 (13.8%)	29/417 (7.0%)	0.034
total HIV positive	109/526 (20.7%)	118/581 (20.3%)	28/103 (27.2%)	90/478 (18.8%)	
Syphilis	n = 529	n = 586	n = 106	n = 480	
tested for syphilis	23/529 (4.3%)	572/586 (97.6%)	102/106 (96.2%)	470/480 (97.9%)	0.302
tested positive	0/23 (0%)	18/572 (3.1%)	2/102 (2%)	16/470 (3.4%)	0.449
Malaria		n = 562	n = 105	n = 457	
tested for mal		554/562 (98.6%)	100/105 (95.2%)	454/457 (99.3%)	0.001
tested positive		177/554 (31.9%)	28/100 (28%)	149/454 (32.8%)	0.349
Anaemia (g/dL)	n = 529	n = 582	n = 106	n = 476	
tested for Hb conc.	147/529 (27.8%)	569/582 (97.8%)	101/106 (95.3%)	468/476 (98.3%)	0.056
Non-anaemic Hb ≥ 10 g/dL	89/147 (60.5%)	346/569 (60.8%)	60/101 (59.4%)	286/468 (61.1%)	
Mild Hb 8–9.9 g/dL	46/147 (31.3%)	166/569 (29.2%)	29/101 (28.7%)	137/468 (29.3%)	
Moderate Hb 5–7.9 g/dL	12/147 (8.2%)	56/569 (9.8%)	11/101 (10.9%)	45/468 (9.6%)	
Severe Hb <5 g/dL	0/147 (0%)	1/569 (0.2%)	1/101 (1%)	0/468 (0%)	0.185
WHO anaemic Hb <11 g/dL	91/147 (61.9%)	346/569 (60.8%)	65/101 (64.4%)	281/468 (60%)	
**Demographics**		**During integrated POCT (n = 586)**			
		All	Not interviewed n = 106	Interviewed n = 480	P-value
Age		n = 585	n = 106	n = 479	
<20		159 (27.2%)	30 (28.3%)	129 (26.9%)	
20 to <25		208 (35.6%)	40 (37.7%)	168 (35.1%)	
25 to <30		118 (20.2%)	18 (17%)	100 (20.9%)	
30 to <35		64 (10.9%)	12 (11.3%)	52 (10.9%)	
35+		36 (6.2%)	6 (5.7%)	30 (6.3%)	0.917*
Gestation in weeks		n = 583	n = 106	n = 477	
median (IQR)		21.1 (16.9–24.1)	21.7 (17.6–24.4)	21.1 (16.9–24.1)	0.47^a^
Gravidity		n = 586	n = 106	n = 480	
gravida 1		163 (27.9%)	38 (35.8%)	125 (26.1%)	
gravida 2		134 (22.9%)	24 (22.6%)	110 (23%)	
gravida 3		114 (19.5%)	14 (13.2%)	100 (20.9%)	
gravida 4		70 (12%)	12 (11.3%)	58 (12.1%)	
gravida 5+		105 (17.9%)	18 (17%)	87 (18.1%)	0.22*
Previous miscarriage		n = 586	n = 106	n = 480	
Yes		21 (3.6%)	0 (0%)	21 (4.4%)	0.028*
Marital status		n = 586	n = 106	n = 480	
married		466 (79.5%)	83 (78.3%)	383 (79.8%)	
widowed		9 (1.5%)	2 (1.9%)	7 (1.5%)	
single		93 (15.9%)	15 (14.2%)	78 (16.3%)	
divorced/separated		18 (17.2%)	6 (5.9%)	12 (12%)	0.363*

WHO Hb: World Health Organization standards for haemoglobin cut-offs for defining anaemia else Kenyan cut-offs

*p-value based on Chi^2^ for the difference between those interviewed and not interviewed

a: p-value based on Wilcoxon rank-sum for the difference in median between those interviewed and not interviewed

ANC: antenatal care; POCT: point-of-care testing; Hb: haemoglobin concentration

### Mixed programme fidelity in management of conditions (self-reported from exit interviews)

Among 586 pregnancies eligible for exit interviews, 106 (18.1%) did not consent. Those interviewed were more likely to have had a previous miscarriage, or have been tested for malaria, and were less likely to be newly diagnosed HIV positive (Table 3).

#### HIV

Of 29 women who were newly tested positive for HIV, treatment was commenced that same visit for less than the 90% target: 19 (65.5%) were given CTX, and 14 (48.3%) were given ARVs (Table 4). One woman was told to return the following day for treatment while seven (24.1%) women refused treatment citing reasons such as: they should consult their husbands; they will start therapy at other clinics; they need to confirm at other clinics; they did not like the nurse’s attitude.

**Table 4:** Self-reported treatments for test positives, IPTp for malaria, and haematinic supplementation given at first visit ANC among 480 interviewed women.

	Proportion	Success threshold	Target reached for correct ANC strategies? *
**HIV treatment**			
Given CTX	19/29 (65.5%)	≥90%	Under-reached
Given ARVs	14/29 (48.3%)	≥90%	Very under-reached
**Syphilis treatment**			
Given 2.4 MU benzathine penicillin or erythromycin	12/17 (70.6%)	≥90%	Under-reached
**Malaria intermittent preventive therapy and treatment**			
Given SP for IPTp among women in 2^nd^ trimester not on CTX†	69/359 (19.2%)	≥90%	Severely under-reached
Given SP for IPTp among women in 1^st^ trimester not on CTX†	5/41 (12.2%)		
Given SP to women also on CTX‡	2/80 (2.5%)		
Given 5 mg folic acid with SP	7/76 (9.2%)		
Given AL, DP or quinine among malaria positives in 2^nd^ trimester	121/153 (79.1%)	≥90%	Under-reached
Given quinine among malaria positives in 1^st^ trimester	2/8 (25%)	≥90%	Severely under-reached
**Haematinic supplementation for prophylaxis and treatment**			
Given folic acid	421/480 (87.7%)	≥90%	Under-reached
- Told to take between 0.4–1.2 mg daily	378/421 (89.8%)		
- Told to take between 5–10 mg daily	43/421 (10%)		
Given iron	434/480 (90.4%)	≥90%	Reached
- Given iron and mentioned side effects	41/434 (9.4%)	≥90%	Severely under-reached
**Iron dosing information given to women according to Hb level**			
Normal Hb ≥10 g/dL	n = 286		
- 60–65 mg elemental iron daily	193/286 (67.4%)	≥90%	Under-reached
- 120–130 mg elemental iron daily	4/286 (1.4%)		
- 180–195 mg elemental iron daily	49/286 (17.1%)		
- Not told dosing	12/286 (4.2%)		
- Not given any iron	28/286 (9.8%)		
Mild anaemia Hb 8–9.9 g/dL	n = 137		
- 60–65 mg elemental iron daily	86/137 (62.8%)		
- 120–130 mg elemental iron daily	4/137 (2.9%)	≥90%	Severely under-reached
- 180–195 mg elemental iron daily	32/137 (23.4%)		
- Not told dosing	3/137 (2.2%)		
- Not given any iron	12/137 (8.8%)		
Moderate anaemia Hb 5–7.9 g/dL	n = 45		
- 60–65 mg elemental iron daily	20/45 (44.4%)		
- 120–130 mg elemental iron daily	9/45 (20%)	≥90%	Severely under-reached
- 180–195 mg elemental iron daily	9/45 (20%)		
- Not told dosing	2/45 (4.4%)		
- Not given any iron	5/45 (11.1%)		
No Hb recorded	n = 12		
- 60–65 mg elemental iron daily	8/12 (66.7%)		
- 120–130 mg elemental iron daily	1/12 (8.3%)		
- Not told dosing	2/12 (16.7%)		
- Not given any iron	1/12 (8.3%)		

* ≥90% target reached, <90% to ≥60—under-reached, <60% to ≥40 very under-reached, <40% severely under-reached

IPTp: intermittent preventive therapy in pregnancy; ANC: antenatal care; CTX: cotrimoxazole; ARV: antiretroviral; SP:sulfadoxine-pyrimethamine; AL: artemether/lumefantrine; DP: dihydroartemisinin-piperaquine (DP); Hb: haemoglobin concentration

†not on CTX includes women who were tested HIV negative, unknown status, and new positive but were not given CTX same day

‡on CTX includes known HIV positives and new HIV positives who were given CTX same day

#### Syphilis

Among 441 women who self-reported receiving syphilis tests, 17 (3.9%) reported positive results of which 12 (70.6%) were given either penicillin (n = 11) or erythromycin (n = 1) the same day (Table 4). One woman was told to return the next day with her partner for couples’ treatment and the remaining four were asked to buy penicillin or erythromycin at a pharmacy even though the study had supplied the facilities with sufficient penicillin based on projected prevalence. Thirteen women (76.5%) said they were advised by the nurse to inform their partners (Table 5).

**Table 5:** Self-reported information given about the tests at first ANC visit among 480 interviewed women.

	Proportion	Success threshold	Target reached for correct ANC strategies?*
**Information about HIV**			
Received HIV counselling	167/480 (34.8%)	≥90%	Severely under-reached
Reported receiving an HIV test among those who had an HIV	411/417 (98.6%)	≥90%	Reached
test result in ANC register			
Reported told HIV status that	399/411 (97%)	≥90%	Reached
agree with result in ANC register			
Reported told HIV status that did not	0/411 (0%)		
agree with result in ANC register			
Reported not told any results for HIV^a^	12/411 (2.9%)		
**Information about syphilis**			
Explained what syphilis is	83/475 (17.5%)	≥90%	Severely under-reached
Reported receiving a syphilis test among those who had a	438/470 (93.2%)	≥90%	Reached
syphilis test result in ANC register			
Reported told syphilis status that	417/438 (95.2%)	≥90%	Reached
agree with result in ANC register			
Reported told syphilis status that did not agree	2/438 (4.6%)		
with result in ANC register			
Reported not told any results for syphilis^a^	19/438 (4.3%)		
Advised to inform partners of syphilis positivity	13/17 (76.5%)	≥90%	Under-reached
**Information about malaria**			
Given advice to use mosquito net to prevent malaria	346/478 (72.4%)	≥90%	Under-reached
Given a mosquito net (n = 349)	331/349 (94.8%)	≥90%	Reached
Reported receiving a malaria test among those who had a	445/454 (98%)	≥90%	Reached
malaria result in ANC register			
Reported told malaria status that	420/445 (94.4%)	≥90%	Reached
agree with result in ANC register			
Reported told malaria status that did not agree	11/445 (2.5%)		
with result in ANC register			
Reported not told any results for malaria^a^	14/445 (3.1%)		
**Information about anaemia**			
Given advice to eat food with iron	132/480 (27.5%)	≥90%	Severely under-reached
Explained what anaemia is	107/480 (22.3%)	≥90%	Severely under-reached
Reported receiving an anaemia test among those who had an	448/468 (95.7%)	≥90%	Reached
Hb result in ANC register			
Reported told an anaemia status that	235/448 (52.5%)	≥90%	Very under-reached
agree with Hb level in ANC register			
Reported told an anaemia status that	60/448 (13.4%)		
did not agree with Hb level in ANC register			
Reported not told any results for anaemia test	153/448 (34.2%)		

* ≥90% target reached, <90% to ≥60 under-reached, <60% to ≥40 very under-reached, <40% severely under-reached

ANC: antenatal care; Hb: haemoglobin concentration; KP: known HIV positives

a: Women reported not told results for HIV, syphilis or malaria were all negative based on ANC registers

#### Treatment and IPTp-SP for malaria

Based on exit interviews, 469 women self-reported receiving malaria tests of which 161 (34.3%) were positive. Among those with positive malaria tests, eight were in their first trimester of whom two (25%) were given quinine and the rest were given or prescribed AL or DP. Of the 153 malaria positive pregnancies in their second trimester, 121 (79.1%) were given AL, DP, or quinine and the rest were given SP (n = 2), prescribed quinine or AL (n = 24) or not given or prescribed anything (n = 6) (Table 4). Therefore 123/161 (76.4%) were given antimalarials in accordance with treatment guidelines.

SP was out of stock for most of the integrated POCT period and only 76 women reported receiving SP for IPTp. Of these, seven were given high dose (5 mg) folic acid concurrently, which is not compliant with guidelines for women receiving IPTp-SP (Table 4). Of the women eligible for SP (in second trimester and not on cotrimoxazole), 19.2% (69/359) received IPTp-SP (Table 4).

#### Anaemia and haematinic supplementation

Among 480 women interviewed, 434 (90.4%) received iron supplementation. However, the reported dosing regimen did not adhere to prophylactic or treatment guidance based on Hb concentrations recorded in the ANC register (Table 4). None of the interviewed women had severe anaemia and six (13.3%) of 45 with moderate anaemia were asked to buy iron dextran. Folic acid was given to 421 (87.7%) women in either 0.4 mg folic-iron combination tablets or 5 mg tablets (Table 4).

#### Information giving

General information and advice given to women about the four conditions is shown in Table 5. Over 90% reported they received the blood tests. Furthermore, over 90% reported test results for HIV, syphilis and malaria that were concordant with those from the ANC register, while only half of those tested for Hb reported anaemia statuses that agreed with those from the register (Table 5). Women who reported not being told results for HIV, syphilis, or malaria were all negative for those conditions based on the ANC register.

### Healthcare worker training, turnover, and performance

Overall 23 healthcare workers (14 nurses: eight females, six males; two clinical officers: two males; six HIV testing counsellors: one female, five males; and one laboratory technician: female) received training and underwent at least one testing QA. All nurses were trained to certificate or diploma level through the government system. Fourteen (60%) of the healthcare workers attended central trainings (eight were trained for five days in November and six for three days in February for those who were unable to make it in November), while nine (40%) received half-day on-site trainings. Turnover of staff was high: of the 16 original healthcare workers at the beginning of the study, only 10 remained by the end of the eight months. Seven new healthcare workers transferred to the facilities during the study and remained until the end. The predictor model suggested that high turnover (adjusted for age, marital status and electricity) was associated with a three-fold increased risk of antenatal women not having a full screening profile (Table 6).

**Table 6:** Predictors of pregnant women not having a full antenatal screening profile at first visits (n = 728).

			Univariate analysis		Multivariate model	
**Individual-level variables**	n = 728	Women without full profile in ANC register	RR (95% CI) of not having a full profile in ANC register	p-value*	RR**(95% CI) of not having a full profile in ANC register	p-value
**Age in years**				0.003		
**≥30**	143 (19.6%)	6 (4.2%)	Reference		Reference	
**≤ 20 to <30**	398 (54.7%)	46 (11.6%)	2.7 (1.5–4.8)		2.9 (1.7–4.9)	<0.0001
**< 20**	187 (25.7%)	18 (9.6%)	2.1 (1.2–3.7)		2.9 (1.6–5.1)	<0.0001
**Marital status *(n = 727)***				0.01		
**single**	116 (16.0%)	6 (5.2%)	Reference		Reference	
**married**	579 (79.6%)	59 (10.2%)	2.4 (1.2–5.0)		2.6 (1.5–4.7)	0.001
**widowed/separated**	32 (4.4%)	5 (15.6%)	3.7 (1.5–9.1)		4.1 (1.7–9.6)	0.001
**Gravidity**				0.89		
**1**	194 (26.7%)	19 (9.8%)	Reference			
**2**	157 (21.6%)	17 (10.8%)	1.1 (0.5–2.4)			
**3+**	377 (51.8%)	34 (9.0%)	0.9 (0.7–1.3)			
**Previous miscarriage**				0.52		
**no**	701 (96.3%)	68 (9.7%)	Reference			
**yes**	27 (3.7%)	2 (7.4%)	0.6 (0.1–2.9)			
**Trimester**				0.35		
**1^st^/2^nd^**	587 (80.6%)	54 (9.2%)	Reference			
**3^rd^**	141 (19.4%)	16 (11.3%)	1.2 (0.8–1.9)			
**Facility-level variables**						
**Staff turnover^a^**				0.0006		
**low**	446 (61.3%)	27 (6.1%)	Reference		Reference	
**medium**	151 (20.7%)	20 (13.3%)	2.2 (0.8–6.1)		1.9 (0.6–5.6)	0.253
**high**	131 (18.0%)	23 (17.6%)	2.9 (1.7–5.0)		3.0 (1.8–5.1)	<0.0001
**Facility volume^b^**				0.4		
**low**	151 (20.7%)	20 (13.2%)	Reference			
**medium**	297 (40.8%)	19 (6.4%)	0.5 (0.2–1.6)			
**high**	280 (38.5%)	31 (11.1%)	0.9 (0.3–2.9)			
**Skilled staff^c^**				0.8		
**2**	206 (28.3%)	16 (7.8%)	Reference			
**3**	167 (22.9%)	19 (11.4%)	1.5 (0.4–5.5)			
**4**	355 (48.8%)	35 (9.9%)	1.2 (0.4–3.3)			
**Electricity**				<0.0001		
**not present**	91 (12.5%)	3 (3.3%)	Reference		Reference	
**present**	637 (87.5%)	67 (10.5%)	3.3 (2.0–5.5)		2.1 (1.2–3.7)	0.01

*Wald test

**n = 727

a: *Staff turnover* was categorized into low, medium and high defined as having 2, 1, and 0 skilled healthcare workers who received training at the start of the programme and remained for all 8 months of implementation respectively

b: *Facility volume* was split into low, medium, and high for <30, 30–40, and 50–70 monthly ANC visits respectively

c: *Skilled staff* was defined as the total number of nurses, clinical officers, and HIV testing counsellors the facility had

ANC: antenatal care; RR: relative risk

**Fig 2:**
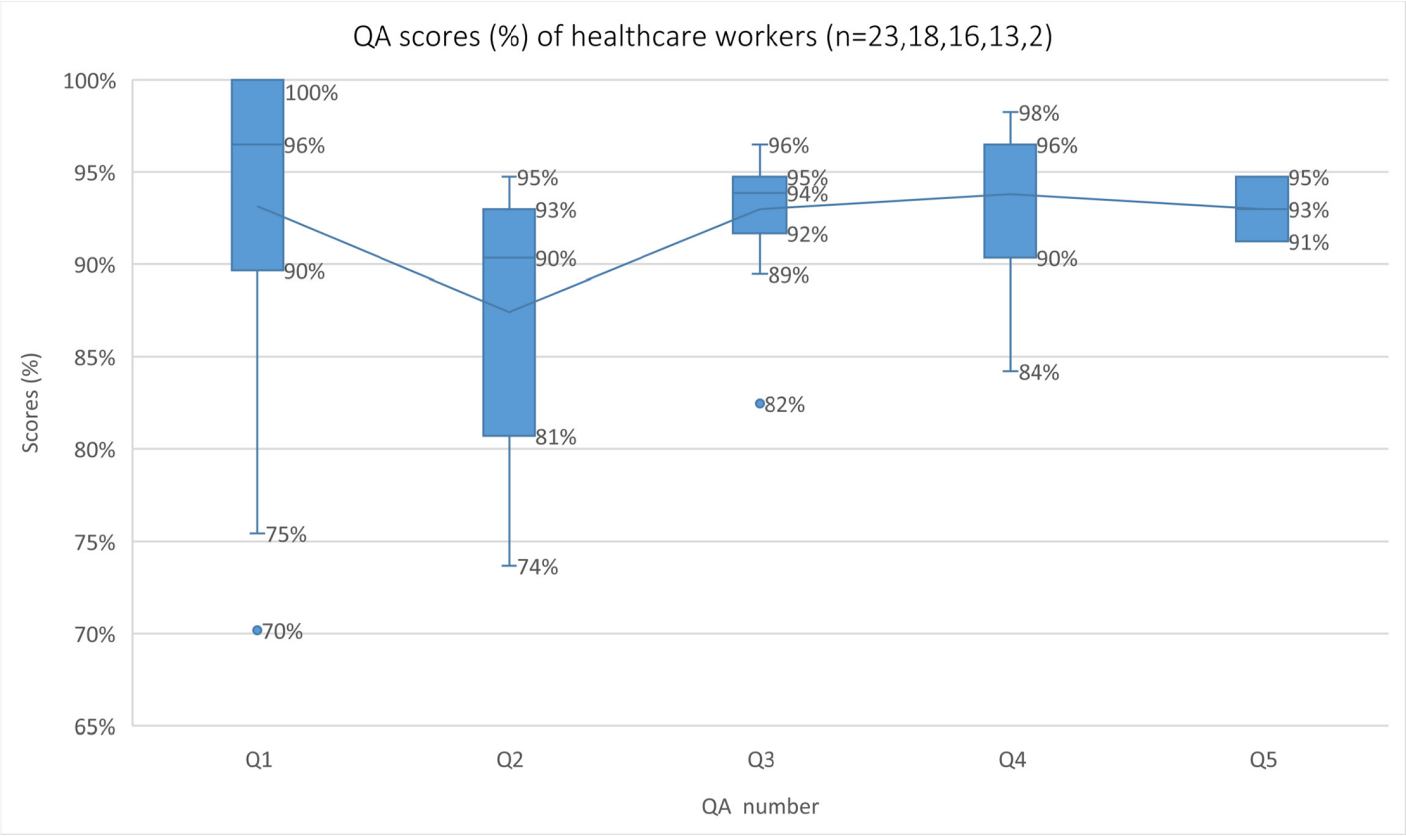
Proficiency scores (%) of healthcare worker checklists in point-of-care testing. The boxes represent interquartile ranges (25% to 75% percentile); the solid line in the box is the median (50th percentile); The lower and upper whiskers represent the minimum and maximum values, excluding outlier; the dots represent outliers, defined as values less than 1.5 times the lower quartile.

Due to turnover and new staff joining, 18 received at least two QAs, 16 received three, 13 received four and two received five. Out of a total of 72 QA scores, 20 (28.6%), belonging to 15 healthcare workers, were below 90% and required more intensive re-training on the same day for steps such as obtaining enough blood from one finger-prick, correctly using the pipette and how to set the timer. Seven of the 15 were evaluated again within a month. Minimum scores improved over the proficiency tests from 70% to 91%. Distribution of scores from the QAs are shown as box and whisker plots in Fig 2. The results of the five QAs suggested that most healthcare workers could accurately conduct integrated POCT after training and remedial training.

## Discussion

This is the first intervention study to evaluate the implementation success of integrating four POCTs into ANC in rural dispensaries in western Kenya. We showed a substantial increase in testing for syphilis, malaria and anaemia that provided an opportunity for the improved management of these conditions in a context of a well-established HIV rapid testing programme in pregnancy. Our results suggest that integrated POCT increases the number of cases detected and treated at the first antenatal visit in these small rural dispensaries without distortion of HIV testing which remained near universal throughout. Despite the management of conditions not reaching our target of 90% fidelity to guidelines, we suspect the treatment for syphilis, malaria and anaemia represented a laudable improvement from baseline. By bringing POCTs to peripheral dispensaries, accessibility and coverage of these tests for pregnant women residing in remote areas, who were otherwise not able to access testing at larger facilities farther away, was significantly improved.

We found prevalence rates of 20.3%, 3.1%, 31.9%, and 60.8% for HIV, syphilis, malaria, and anaemia (WHO cut-off Hb <11 g/dL, or 39.2% when using Kenyan guideline cut-offs of <10 g/dL) respectively, consistent with those reported in other studies [33, 39, 40, 54, 55]. Siaya is about 1,200 meters above sea level and the altitude adjusted prevalence of anaemia using the WHO cut-off was 70% [56].

The success of near universal HIV screening in ANC in Kenya, as reflected in our baseline and intervention data, is the result of concerted vertical program efforts and external partnerships [57–59]. Although WHO has endorsed an integrated disease approach to service delivery of antenatal care [60, 61], development assistance for HIV activities has risen disproportionately since 2000 compared to assistance for other sexual reproductive health activities [62], shifting agendas and priorities away from strengthening systems and building linkages between programmes [44, 59, 63–65]. Funds are often earmarked for specific purposes with deliverables defined by coverage and uptake, creating strong incentives for focused vertical programmes that result in rapid outputs rather than overall health system improvement [63, 66, 67]. In Kenya, less than half of the pregnant women attending ANC are tested for syphilis or haemoglobin concentrations [37–39, 68], even though antenatal screening is a major country policy and over 90% of pregnant women attend ANC [40]. These gaps can likely be reduced by combining funding to support comprehensive ANC programmes and better coordination mechanisms at national and county levels among the National AIDS and STI Control Programme, the Department for Reproductive Health and the National Malaria Control Programme [42, 69]. For malaria, there is currently no national recommendation for screening at the first antenatal clinic visit, but this is commonly practiced in most of the larger facilities with laboratories in the highly malaria endemic areas in western Kenya. Furthermore, there is an increasing interest in this hybrid strategy that combines IPTp-SP with testing at the first visit because of concerns about missed opportunities with IPTp and increasing SP resistance.

A quarter of newly diagnosed HIV positive women declined immediate treatment. This low uptake of ARVs is consistent with the finding of a meta-analysis in 2012 that reported only 73.5% of pregnant women in low, middle and high-income countries achieved optimal adherence to ARV therapy during and after pregnancy [70]. Quality of HIV counselling, lack of emphasis of the importance of ARV adherence at post-test counselling, lack of male involvement, and lack of trust of ANC staff are reasons reported to be associated with low adherence [70]. In our study, we found low HIV counselling rates, which could explain why many women were unprepared for positive results [71]. Dosing of iron among healthcare workers at the seven dispensaries was not consistent and did not follow the national guidelines for iron supplementation. These inconsistencies have also been highlighted in government surveys conducted in 2013 which reported a lack of consistency in implementation of haematinic supplementation at health facilities, poor knowledge of dosing and duration, inadequate knowledge given to pregnant women about anaemia, low adherence and compliance, and limited understanding of side effects leading to discontinuation [68]. The uptake of IPTp-SP was very low or the intervention was incorrectly implemented, mostly because of stock-outs and poor understanding of guidelines, falling well short of the target for 80% coverage by 2016 [72]. Poor IPTp-SP program implementation by health providers, stemming from unclear guidelines, stock-outs, poor facility organization, and low provider knowledge, had been reported elsewhere [36, 73]. Case-management of malaria test-positive cases was much better than IPTp-SP coverage, with almost 80% of women receiving treatment with an ACT or quinine. Consistent with a previous study in this area by Riley et al [74], adherence to the national case-management guidelines for women in the first trimester was inadequate and most women were prescribed ACTs instead of the first line treatment with quinine. Starting in 2013 and during the integrated POCT study period, Kenya was devolving the healthcare system and transferring authority for decision making, finance and management of health commodities to the county level. This resulted in a disruption of the malaria commodity supply chain during this transition period and corresponding stock-outs of SP and antimalarials, which may explain some of these findings.

Ensuring all healthcare workers were trained to standard and have quality maintained over time was challenging. High turnover of staff meant on-site training of new staff would pose challenges if the intervention were to scale in real-life conditions unless training for POCTs were incorporated into nursing school or was done on a country-level. Small, focused trainings with interactions are more effective than didactic ones and will require time and planning [75]. Since healthcare worker behaviours are complex with many contextual and environmental influencers, single, one-off interventions such as trainings or dissemination of guidelines are less effective than routine supervisions and feedback to maintain high-quality performance [75]. Quality improvement (QI) strategies that are step-wise, iterative, locally grown and adapted to existing systems may be more appropriate for long-term sustainability but context specific strategies need to be cultivated and effectiveness monitored in real-time [75–77].

There are several limitations to our study. We did not randomly sample the facilities and therefore they may not be representative of all tier-2 facilities in the region. The evaluation excluded women who attended their first ANC visits in the third trimester, which was about 20% of first ANC attendees. While they also received the same services as first and second trimester women, their late attendance precludes early management of conditions and interventions would be needed to address this population. We used routinely collected ANC register data to calculate testing uptakebut there were inconsistencies in how the registers were filled. For example, there were no columns for recording malaria test results in the existing ANC register and so we hand-drew a column during the study period. This was not always communicated to rotating ANC staff which resulted in 23 missing records for malaria testing. ANC registers do not record treatment and so we relied on interviews with women to obtain information on treatments and there were minor incongruences between self-reported and register recorded test results. More effort is needed to ensure accurate record keeping. Exit interviews were done with women at the facility immediately after an ANC visit; while this minimizes recall bias, women may be less inclined to report on negative experiences or give perceived acceptable responses without having understood the questions fully (courtesy bias). We did not assess the continuum of clinical management fidelity at revisits or at delivery. For example, new-borns of women given erythromycin for syphilis need to be treated immediately with a regimen of penicillin injections [78] but this was not measured. Effort would be needed to ensure healthcare worker’s understanding and adherence to clinical management *throughout* the pregnancy and perinatal period. This study assessed clinical practice at the service delivery stage of the implementation pathway [43] where the conditions of having test supplies available, training and supervision were met through provision by the study. To effect positive health outcomes, wider health system concerns of supply chains, stock-outs and human resource management would need to be addressed.

## Conclusion

This study showed near universal HIV testing in rural dispensaries that lack laboratory facilities and increased uptake of testing for syphilis, malaria and anaemia following training and availability of POCTs. However, poor clinical management of conditions, frequent staff turnover and inadequate information given to pregnant women remain significant challenges that will require integrated funding channels and a robust quality assurance programme to ensure that standards are achieved and maintained in these peripheral facilities.

## Supporting information

S1 Fig Integration operating procedure placemat. (TIF)

S1 Text Proficiency testing monitoring checklist. (DOCX)

## References

[1] Zaba B, Calvert C, Marston M, Isingo R, Nakiyingi-Miiro J, Lutalo T, et al Effect of HIV infection on pregnancy-related mortality in sub-Saharan Africa: secondary analyses of pooled community-based data from the network for Analysing Longitudinal Population-based HIV/AIDS data on Africa (ALPHA). Lancet. 2013;381(9879):1763–71. 10.1016/S0140-6736(13)60803-X ; PubMed Central PMCID: PMCPMC4325135. 23683643 PMC4325135

[2] Wijesooriya NS, Rochat RW, Kamb ML, Turlapati P, Temmerman M, Broutet N, et al Global burden of maternal and congenital syphilis in 2008 and 2012: a health systems modelling study. Lancet Glob Health. 2016;4(8):e525–33. 10.1016/S2214-109X(16)30135-8 . 27443780 PMC6759483

[3] Desai M, ter Kuile FO, Nosten F, McGready R, Asamoa K, Brabin B, et al Epidemiology and burden of malaria in pregnancy. The Lancet infectious diseases. 2007;7(2):93–104. 10.1016/S1473-3099(07)70021-X . 17251080

[4] Stevens GA, Finucane MM, De-Regil LM, Paciorek CJ, Flaxman SR, Branca F, et al Global, regional, and national trends in haemoglobin concentration and prevalence of total and severe anaemia in children and pregnant and non-pregnant women for 1995–2011: a systematic analysis of population-representative data. Lancet Glob Health. 2013;1(1):e16–25. 10.1016/S2214-109X(13)70001-9 ; PubMed Central PMCID: PMCPMC4547326. 25103581 PMC4547326

[5] Lynn WA, Lightman S. Syphilis and HIV: a dangerous combination. The Lancet infectious diseases. 2004;4(7):456–66. 10.1016/S1473-3099(04)01061-8 . 15219556

[6] Gonzalez R, Ataide R, Naniche D, Menendez C, Mayor A. HIV and malaria interactions: where do we stand? Expert Rev Anti Infect Ther. 2012;(2):153 10.1586/eri.11.167 22339190

[7] Chico R, Mayaud P, Ariti C, Mabey D, Ronsmans C, Chandramohan D. Prevalence of malaria and sexually transmitted and reproductive tract infections in pregnancy in sub-saharan africa: A systematic review. JAMA. 2012;307(19):2079–86. 10.1001/jama.2012.3428 22665107

[8] Guyatt HL, Snow RW. The epidemiology and burden of Plasmodium falciparum-related anemia among pregnant women in sub-Saharan Africa. The American journal of tropical medicine and hygiene. 2001;64(1–2 Suppl):36–44. . 11425176 10.4269/ajtmh.2001.64.36

[9] Orish VN, Onyeabor OS, Boampong JN, Acquah S, Sanyaolu AO, Iriemenam NC. The effects of malaria and HIV co-infection on hemoglobin levels among pregnant women in Sekondi-Takoradi, Ghana. International journal of gynaecology and obstetrics: the official organ of the International Federation of Gynaecology and Obstetrics. 2013;120(3):236–9. 10.1016/j.ijgo.2012.09.021 . 23219288

[10] Eaton JW, Rehle TM, Jooste S, Nkambule R, Kim AA, Mahy M, et al Recent HIV prevalence trends among pregnant women and all women in sub-Saharan Africa: implications for HIV estimates. Aids. 2014;28 Suppl 4:S507–14. Epub 2014/11/20. 10.1097/qad.0000000000000412 ; PubMed Central PMCID: PMCPMC4247272. 25406753 PMC4247272

[11] The Joint United Nations Programme on HIV/AIDS (UNAIDS). Regional Fact Sheet 2012: Sub-Saharan Africa. Geneva, Switzerland: 2012.

[12] Dabis F, Ekpini ER. HIV-1/AIDS and maternal and child health in Africa. Lancet. 2002;359(9323):2097–104. 10.1016/S0140-6736(02)08909-2 . 12086778

[13] Fiumara NJ. Syphilis in newborn children. Clin Obstet Gynecol. 1975;18(1):183–9. . 1091383 10.1097/00003081-197503000-00016

[14] Gomez GB, Kamb ML, Newman LM, Mark J, Broutet N, Hawkes SJ. Untreated maternal syphilis and adverse outcomes of pregnancy: a systematic review and meta-analysis. Bulletin of the World Health Organization. 2013;91(3):217–26. 10.2471/BLT.12.107623 ; PubMed Central PMCID: PMC3590617. 23476094 PMC3590617

[15] Walker PG, ter Kuile FO, Garske T, Menendez C, Ghani AC. Estimated risk of placental infection and low birthweight attributable to Plasmodium falciparum malaria in Africa in 2010: a modelling study. Lancet Glob Health. 2014;2(8):e460–7. 10.1016/S2214-109X(14)70256-6 . 25103519

[16] Steketee RW, Nahlen BL, Parise ME, Menendez C. The burden of malaria in pregnancy in malaria-endemic areas. The American journal of tropical medicine and hygiene. 2001;64(1–2 Suppl):28–35. PubMed PMID: 11425158. 10.4269/ajtmh.2001.64.28 11425175

[17] Brabin BJ. An analysis of malaria in pregnancy in Africa. Bulletin of the World Health Organization. 1983;61(6):1005–16. . 6370484 PMC2536236

[18] Rogerson SJ, Desai M, Mayor A, Sicuri E, Taylor SM, van Eijk AM. Burden, pathology, and costs of malaria in pregnancy: new developments for an old problem. The Lancet Infectious Diseases. 2018 10.1016/S1473-3099(18)30066-5 . 29396010

[19] Huynh B-T, Cottrell G, Cot M, Briand V. Burden of Malaria in Early Pregnancy: A Neglected Problem? Clinical Infectious Diseases. 2015;60(4):598–604. 10.1093/cid/ciu848 25362205

[20] World Health Organization. The global prevalence of anaemia in 2011. Geneva, Switzerland: World Health Organization, 2015.

[21] Stoltzfus RJ. Iron interventions for women and children in low-income countries. J Nutr. 2011;141(4):756S–62S. 10.3945/jn.110.128793 . 21367936

[22] Kavle JA, Stoltzfus RJ, Witter F, Tielsch JM, Khalfan SS, Caulfield LE. Association between Anaemia during Pregnancy and Blood Loss at and after Delivery among Women with Vaginal Births in Pemba Island, Zanzibar, Tanzania. Journal of Health, Population, and Nutrition. 2008;26(2):232–40. PubMed PMID: PMC2740668. 18686556 PMC2740668

[23] Allen LH. Anemia and iron deficiency: effects on pregnancy outcome. Am J Clin Nutr. 2000;71(5 Suppl):1280S–4S. 10.1093/ajcn/71.5.1280s . 10799402

[24] Ramakrishnan U, Grant F, Goldenberg T, Zongrone A, Martorell R. Effect of women's nutrition before and during early pregnancy on maternal and infant outcomes: a systematic review. Paediatric and perinatal epidemiology. 2012;26 Suppl 1:285–301. Epub 2012/07/07. 10.1111/j.1365-3016.2012.01281.x . 22742616

[25] Cuadros DF, Branscum AJ, Crowley PH. HIV-malaria co-infection: effects of malaria on the prevalence of HIV in East sub-Saharan Africa. International journal of epidemiology. 2011;40(4):931–9. 10.1093/ije/dyq256 . 21224274

[26] ter Kuile FO, Parise ME, Verhoeff FH, Udhayakumar V, Newman RD, van Eijk AM, et al The burden of co-infection with human immunodeficiency virus type 1 and malaria in pregnant women in sub-saharan Africa. The American journal of tropical medicine and hygiene. 2004;71(2 Suppl):41–54. . 15331818

[27] Abu-Raddad LJ, Patnaik P, Kublin JG. Dual infection with HIV and malaria fuels the spread of both diseases in sub-Saharan Africa. Science (New York, NY). 2006;314(5805):1603–6. 10.1126/science.1132338 . 17158329

[28] Menendez C, Fleming AF, Alonso PL. Malaria-related anaemia. Parasitol Today. 2000;16(11):469–76. . 11063857 10.1016/s0169-4758(00)01774-9

[29] World Health Organization. WHO recommendations on antenatal care for a positive pregnancy experience. Geneva, Switzerland: 2016. 28079998

[30] The United States Agency for International Development (USAID) Population Council. Acceptability and Sustainability of the WHO Focused Antenatal Care package in Kenya. Washington DC: USAID 2006.

[31] Tagbor H, Bruce J, Agbo M, Greenwood B, Chandramohan D. Intermittent screening and treatment versus intermittent preventive treatment of malaria in pregnancy: a randomised controlled non-inferiority trial. PloS one. 2010;5(12):e14425 10.1371/journal.pone.0014425 ; PubMed Central PMCID: PMCPMC3010999. 21203389 PMC3010999

[32] Tagbor H, Cairns M, Bojang K, Coulibaly SO, Kayentao K, Williams J, et al A Non-Inferiority, Individually Randomized Trial of Intermittent Screening and Treatment versus Intermittent Preventive Treatment in the Control of Malaria in Pregnancy. PloS one. 2015;10(8):e0132247 10.1371/journal.pone.0132247 ; PubMed Central PMCID: PMCPMC4530893. 26258474 PMC4530893

[33] Desai M, Gutman J, L'Lanziva A, Otieno K, Juma E, Kariuki S, et al Intermittent screening and treatment or intermittent preventive treatment with dihydroartemisinin-piperaquine versus intermittent preventive treatment with sulfadoxine-pyrimethamine for the control of malaria during pregnancy in western Kenya: an open-label, three-group, randomised controlled superiority trial. Lancet. 2015;386(10012):2507–19. 10.1016/S0140-6736(15)00310-4 ; PubMed Central PMCID: PMCPMC4718402. 26429700 PMC4718402

[34] Desai M, Gutman J, Taylor SM, Wiegand RE, Khairallah C, Kayentao K, et al Impact of Sulfadoxine-Pyrimethamine Resistance on Effectiveness of Intermittent Preventive Therapy for Malaria in Pregnancy at Clearing Infections and Preventing Low Birth Weight. Clinical infectious diseases: an official publication of the Infectious Diseases Society of America. 2016;62(3):323–33. 10.1093/cid/civ881 ; PubMed Central PMCID: PMCPMC4762476. 26486699 PMC4762476

[35] Chico RM, Chandramohan D. Intermittent preventive treatment of malaria in pregnancy: at the crossroads of public health policy. Tropical medicine & international health: TM & IH. 2011;16(7):774–85. 10.1111/j.1365-3156.2011.02765.x . 21477099

[36] Hill J, Dellicour S, Bruce J, Ouma P, Smedley J, Otieno P, et al Effectiveness of antenatal clinics to deliver intermittent preventive treatment and insecticide treated nets for the control of malaria in pregnancy in Kenya. PloS one. 2013;8(6):e64913 10.1371/journal.pone.0064913 ; PubMed Central PMCID: PMCPMC3683044. 23798997 PMC3683044

[37] van Eijk AM, Bles HM, Odhiambo F, Ayisi JG, Blokland IE, Rosen DH, et al Use of antenatal services and delivery care among women in rural western Kenya: a community based survey. Reproductive health. 2006;3(1):2 10.1186/1742-4755-3-2 ; PubMed Central PMCID: PMCPMC1459114. 16597344 PMC1459114

[38] Ouma PO, van Eijk AM, Hamel MJ, Sikuku ES, Odhiambo FO, Munguti KM, et al Antenatal and delivery care in rural western Kenya: the effect of training health care workers to provide "focused antenatal care". Reproductive health. 2010;7:1 10.1186/1742-4755-7-1 ; PubMed Central PMCID: PMC2867783. 20429906 PMC2867783

[39] Eleanor Fleming JO, Katherine O’Connor, Aloyce Odhiambo, Ye Tun, Simon Oswago CZ, Robert Quick, Kamb Mary L. The Impact of Integration of Rapid Syphilis Testing during Routine Antenatal Services in Rural Kenya. Journal of Sexually Transmitted Diseases 2013. 10.1155/2013/674584 PMC4437431 26316963

[40] National AIDS and STI Control Programme (NASCOP). Kenya AIDS Indicator Survey 2012: Final Report. 2014.

[41] Peeling RW, Mabey D. Point-of-care tests for diagnosing infections in the developing world. Clin Microbiol Infect. 2010;16(8):1062–9. 10.1111/j.1469-0691.2010.03279.x . 20670288

[42] Newman Owiredu M, Newman L, Nzomo T, Conombo Kafando G, Sanni S, Shaffer N, et al Elimination of mother-to-child transmission of HIV and syphilis: A dual approach in the African Region to improve quality of antenatal care and integrated disease control. International journal of gynaecology and obstetrics: the official organ of the International Federation of Gynaecology and Obstetrics. 2015;130 Suppl 1:S27–31. 10.1016/j.ijgo.2015.04.010 . 25963908

[43] Baker U, Okuga M, Waiswa P, Manzi F, Peterson S, Hanson C, et al Bottlenecks in the implementation of essential screening tests in antenatal care: Syphilis, HIV, and anemia testing in rural Tanzania and Uganda. International journal of gynaecology and obstetrics: the official organ of the International Federation of Gynaecology and Obstetrics. 2015;130 Suppl 1:S43–50. 10.1016/j.ijgo.2015.04.017 . 26054252

[44] Gloyd S, Chai S, Mercer MA. Antenatal syphilis in sub-Saharan Africa: missed opportunities for mortality reduction. Health policy and planning. 2001;16(1):29–34. . 11238427 10.1093/heapol/16.1.29

[45] Mallma P, Garcia P, Carcamo C, Torres-Rueda S, Peeling R, Mabey D, et al Rapid Syphilis Testing Is Cost-Effective Even in Low-Prevalence Settings: The CISNE-PERU Experience. PloS one. 2016;11(3):e0149568 10.1371/journal.pone.0149568 ; PubMed Central PMCID: PMCPMC4780822. 26949941 PMC4780822

[46] Jafari Y, Peeling RW, Shivkumar S, Claessens C, Joseph L, Pai NP. Are Treponema pallidum specific rapid and point-of-care tests for syphilis accurate enough for screening in resource limited settings? Evidence from a meta-analysis. PloS one. 2013;8(2):e54695 Epub 2013/03/08. 10.1371/journal.pone.0054695 ; PubMed Central PMCID: PMCPmc3582640. 23468842 PMC3582640

[47] Medina Lara A MC, Kandulu J, Chisuwo L, Bates I,. Evaluation and costs of different haemoglobin methods for use in district hospitals in Malawi. Journal of Clinical Pathology. 2005;58(1):56–60. 10.1136/jcp.2004.018366 15623483 PMC1770538

[48] Odhiambo FO, Laserson KF, Sewe M, Hamel MJ, Feikin DR, Adazu K, et al Profile: the KEMRI/CDC Health and Demographic Surveillance System—Western Kenya. International journal of epidemiology. 2012;41(4):977–87. 10.1093/ije/dys108 . 22933646 PMC12083774

[49] The United States Agency for International Development. The Demographic and Health Surveys Program [cited 2017 March 19]. Available from: http://www.dhsprogram.com/What-We-Do/Survey-Types/SPA.cfm.

[50] Ministry of Health Kenya. National Guidelines for Quality Obstetrics and Perinatal Care. Nairobi, Kenya: Ministry of Health, 2012.

[51] Proctor E, Silmere H, Raghavan R, Hovmand P, Aarons G, Bunger A, et al Outcomes for implementation research: conceptual distinctions, measurement challenges, and research agenda. Adm Policy Ment Health. 2011;38(2):65–76. 10.1007/s10488-010-0319-7 ; PubMed Central PMCID: PMCPMC3068522. 20957426 PMC3068522

[52] The Joint United Nations Programme on HIV/AIDS (UNAIDS). Fast-track: accelerating action to end the AIDS epidemic by 2030. Geneva, Switzerland: 2015.

[53] World Health Organization. The global elimination of congenital syphilis: rationale and strategy for action. Geneva, Switzerland: 2007.

[54] Otieno-Nyunya B, Bennett E, Bunnell R, Dadabhai S, Gichangi AA, Mugo N, et al Epidemiology of syphilis in Kenya: results from a nationally representative serological survey. Sexually transmitted infections. 2011;87(6):521–5. Epub 2011/09/16. 10.1136/sextrans-2011-050026 . 21917697

[55] Turan JM, Steinfeld RL, Onono M, Bukusi EA, Woods M, Shade SB, et al The study of HIV and antenatal care integration in pregnancy in Kenya: design, methods, and baseline results of a cluster-randomized controlled trial. PloS one. 2012;7(9):e44181 Epub 2012/09/13. 10.1371/journal.pone.0044181 ; PubMed Central PMCID: PMCPmc3435393. 22970177 PMC3435393

[56] World Health Organization. Haemoglobin concentrations for the diagnosis of anaemia and assessment of severity. Geneva, World Health Organization: 2011.

[57] Marum E, Taegtmeyer M, Parekh B, Mugo N, Lembariti S, Phiri M, et al "What took you so long?" The impact of PEPFAR on the expansion of HIV testing and counseling services in Africa. Journal of acquired immune deficiency syndromes (1999). 2012;60 Suppl 3:S63–9. 10.1097/QAI.0b013e31825f313b . 22797742

[58] Marum E, Taegtmeyer M, Chebet K. Scale-up of voluntary hiv counseling and testing in kenya. JAMA. 2006;296(7):859–62. 10.1001/jama.296.7.859 16905791

[59] Druce N, Nolan A. Seizing the big missed opportunity: linking HIV and maternity care services in sub-Saharan Africa. Reprod Health Matters. 2007;15(30):190–201. 10.1016/S0968-8080(07)30337-6 . 17938084

[60] Kerber KJ, de Graft-Johnson JE, Bhutta ZA, Okong P, Starrs A, Lawn JE. Continuum of care for maternal, newborn, and child health: from slogan to service delivery. Lancet. 2007;370(9595):1358–69. 10.1016/S0140-6736(07)61578-5 . 17933651

[61] World Health Organization. Integrated health services- what and why? Technical brief No. 1 Geneva, Switzerland: World Health Organization; 5 2008 [cited 2017 May 2017]. Available from: http://www.who.int/healthsystems/technical_brief_final.pdf.

[62] Dieleman JL, Schneider MT, Haakenstad A, Singh L, Sadat N, Birger M, et al Development assistance for health: past trends, associations, and the future of international financial flows for health. Lancet. 2016;387(10037):2536–44. 10.1016/S0140-6736(16)30168-4 . 27086170

[63] Druce N, Dickinson C, Attawell K, Campbell AW, Standing H. Strengthening linkages for sexual and reproductive health, HIV and AIDS: progress, barriers and opportunities for scaling up London, UK: Health Resource Centre, British Government’s Department for International Development (DFID); 2006 [March 17, 2017]. Available from: http://hdrc.dfid.gov.uk/wpcontent/uploads/2012/10/Strenghtening-linkages-for-sexual-and-reproductivehealth.pdf.

[64] Grepin KA. HIV donor funding has both boosted and curbed the delivery of different non-HIV health services in sub-Saharan Africa. Health Aff (Millwood). 2012;31(7):1406–14. 10.1377/hlthaff.2012.0279 . 22778329

[65] Peeling RW, Mabey D, Fitzgerald DW, Watson-Jones D. Avoiding HIV and dying of syphilis. Lancet. 2004;364(9445):1561–3. 10.1016/S0140-6736(04)17327-3 . 15519615

[66] Fowkes FJ, Draper BL, Hellard M, Stoove M. Achieving development goals for HIV, tuberculosis and malaria in sub-Saharan Africa through integrated antenatal care: barriers and challenges. BMC Med. 2016;14(1):202 10.1186/s12916-016-0753-9 ; PubMed Central PMCID: PMCPMC5151135. 27938369 PMC5151135

[67] de Jongh TE, Gurol-Urganci I, Allen E, Jiayue Zhu N, Atun R. Barriers and enablers to integrating maternal and child health services to antenatal care in low and middle income countries. BJOG. 2016;123(4):549–57. 10.1111/1471-0528.13898 ; PubMed Central PMCID: PMCPMC4768640. 26861695 PMC4768640

[68] Ministry of Health Kenya. National Iron and Folic Acid Supplementation Communication Strategy. Nairobi, Kenya: Ministry of Health, 2013.

[69] Ministry of Health Kenya. Kenya Reproductive, Maternal, Newborn, Child and Adolescent Health (RMNCAH) Investment Framework. Nairobi, Kenya: Ministry of Health, 2016.

[70] Nachega JB, Uthman OA, Anderson J, Peltzer K, Wampold S, Cotton MF, et al Adherence to antiretroviral therapy during and after pregnancy in low-income, middle-income, and high-income countries: a systematic review and meta-analysis. Aids. 2012;26(16):2039–52. 10.1097/QAD.0b013e328359590f ; PubMed Central PMCID: PMCPMC5061936. 22951634 PMC5061936

[71] Colombini M, Stockl H, Watts C, Zimmerman C, Agamasu E, Mayhew SH. Factors affecting adherence to short-course ARV prophylaxis for preventing mother-to-child transmission of HIV in sub-Saharan Africa: a review and lessons for future elimination. AIDS care. 2014;26(7):914–26. 10.1080/09540121.2013.869539 . 24354642

[72] The United States Agency for International Development (USAID) President's Malaria Initiative (PMI). Malaria Operational Plan FY 2017. 2017.

[73] Hill J, Hoyt J, van Eijk AM, D'Mello-Guyett L, Ter Kuile FO, Steketee R, et al Factors affecting the delivery, access, and use of interventions to prevent malaria in pregnancy in sub-Saharan Africa: a systematic review and meta-analysis. PLoS medicine. 2013;10(7):e1001488 10.1371/journal.pmed.1001488 ; PubMed Central PMCID: PMCPMC3720261. 23935459 PMC3720261

[74] Riley C, Dellicour S, Ouma P, Kioko U, ter Kuile FO, Omar A, et al Knowledge and Adherence to the National Guidelines for Malaria Case Management in Pregnancy among Healthcare Providers and Drug Outlet Dispensers in Rural, Western Kenya. PloS one. 2016;11(1):e0145616 10.1371/journal.pone.0145616 ; PubMed Central PMCID: PMCPMC4720358. 26789638 PMC4720358

[75] Rowe AK, de Savigny D, Lanata CF, Victora CG. How can we achieve and maintain high-quality performance of health workers in low-resource settings? Lancet. 2005;366(9490):1026–35. 10.1016/S0140-6736(05)67028-6 . 16168785

[76] Berwick DM. Developing and testing changes in delivery of care. Ann Intern Med. 1998;128(8):651–6. . 9537939 10.7326/0003-4819-128-8-199804150-00009

[77] Heiby J. The use of modern quality improvement approaches to strengthen African health systems: a 5-year agenda. Int J Qual Health Care. 2014;26(2):117–23. 10.1093/intqhc/mzt093 . 24481053

[78] World Health Organization. WHO guidlines for the treatment of treponema pallidum (syphilis). Geneva, Switzerland: 2016. 27631046

