# Supplementary figures and images for: Integrated point-of-care testing (POCT) of HIV, syphilis, malaria and anaemia in antenatal clinics in western Kenya: A longitudinal implementation study
Source: PLoS One. 2018 Jul 20;13(7):e0198784. doi: 10.1371/journal.pone.0198784 (PMC6054376; doi:10.1371/journal.pone.0198784)

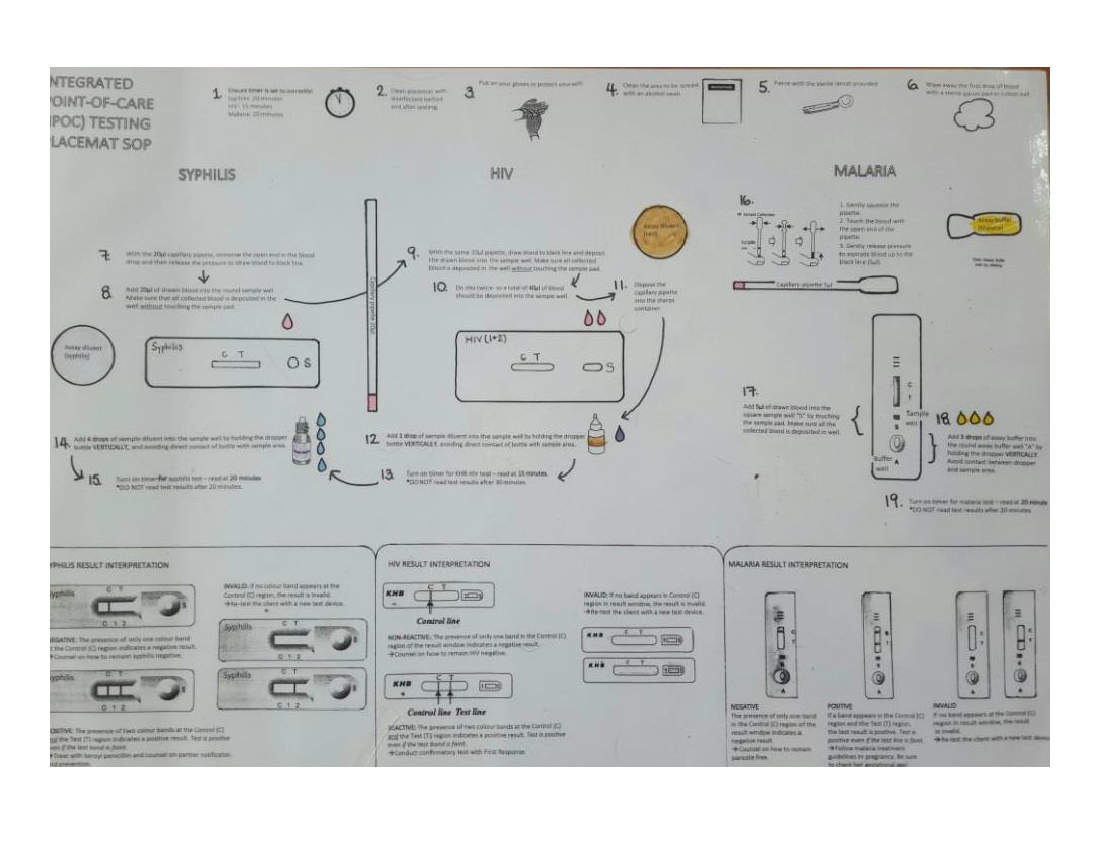

Supplement: S1 Fig — (TIF) [file pone.0198784.s001.tif]
