# Supplementary material for: Integrated point-of-care testing (POCT) of HIV, syphilis, malaria and anaemia in antenatal clinics in western Kenya: A longitudinal implementation study
Source: PLoS One. 2018 Jul 20;13(7):e0198784. doi: 10.1371/journal.pone.0198784 (PMC6054376; doi:10.1371/journal.pone.0198784)
Supplement: S1 Text — (DOCX) [file pone.0198784.s002.docx]

# Proficiency testing monitoring checklist

IPOCT EXTERNAL MONITORING CHECKLIST

Name of facility:

Name of staff:

Date:

|  |  | **Y** | **N** |  | **Y** | **N** |
| --- | --- | --- | --- | --- | --- | --- |
| **Safety:** | Sharps container | □ | □ | Gloves | □ | □ |
|  | Biohazard waste bin/bag | □ | □ |  |  |  |

**PERFORMANCE OF PROCEDURES**

|  |  | **Y** | **N** |  | **Y** | **N** |
| --- | --- | --- | --- | --- | --- | --- |
| **Preparation** | Label initials onto cassette | □ | □ | Check lot number | □ | □ |
|  | Correct use of timer | □ | □ | Check expiration date | □ | □ |
| **Finger prick** | Sterile lancet | □ | □ | Correct position of prick | □ | □ |
|  | Correct massage | □ | □ | Dispose lancet in sharps | □ | □ |
|  | Correct disinfection | □ | □ | Correct wiping of first drop | □ | □ |
|  |  |  |  | Sufficient volume of blood for IPOCT using 1 prick (4 tests) | □ | □ |

|  |  | **Y** | **N** |  | **Y** | **N** |
| --- | --- | --- | --- | --- | --- | --- |
| **Syphilis Testing:** | Syphilis pipette | □ | □ | Deposit all blood onto sample well *without* touching sample pad | □ | □ |
|  | Syphilis buffer | □ | □ | Dispose pipette in sharps | □ | □ |
|  | Syphilis cassette | □ | □ | Correct number of buffer drops into sample well | □ | □ |
|  | Correct pipetting | □ | □ | Starting timer immed’ly after buffer | □ | □ |
|  | Sufficient volume drawn to line (20µl) | □ | □ | Waiting full time to read -ve result | □ | □ |
|  |  |  |  | Correctly interpret result | □ | □ |
|  |  |  |  |  |  |  |
|  |  | **Y** | **N** |  | **Y** | **N** |
| **HIV Testing:** | HIV pipette | □ | □ | Deposit all blood onto sample well *without* touching sample pad | □ | □ |
|  | HIV buffer | □ | □ | Dispose pipette in sharps | □ | □ |
|  | HIV cassette | □ | □ | Correct number of buffer drops into sample well | □ | □ |
|  | Correct pipetting | □ | □ | Starting timer immed’ly after buffer | □ | □ |
|  | Sufficient volume drawn to line (40µl) | □ | □ | Waiting full time to read -ve result | □ | □ |
|  |  |  |  | Correctly interpret result | □ | □ |
|  |  |  |  |  |  |  |
|  |  | **Y** | **N** |  | **Y** | **N** |
| **Malaria Testing:** | Malaria pipette | □ | □ | Deposit all blood onto sample well *touching* **sample pad** | □ | □ |
|  | Malaria buffer | □ | □ | Dispose pipette in sharps | □ | □ |
|  | Malaria cassette | □ | □ | Correct number of buffer drops into **buffer well** | □ | □ |
|  | Correct pipetting | □ | □ | Starting timer immed’ly after buffer | □ | □ |
|  | Sufficient volume drawn to line (5µl) |  |  | Waiting full time to read -ve result | □ | □ |
|  |  |  |  | Correctly interpret result | □ | □ |
|  |  |  |  |  |  |  |
|  |  | **Y** | **N** |  | **Y** | **N** |
| **HB testing:** | Hemocuvettes | □ | □ | Correct wiping of cuvette on both sides | □ | □ |
|  | Hemocue machine | □ | □ | *Gently* closing machine | □ | □ |
|  | Kimwipe | □ | □ | Correctly reading result | □ | □ |
|  | Correctly turn on hemocue machine | □ | □ | Taking cuvette out of machine | □ | □ |
|  | Correct drawing of blood into cuvette | □ | □ | Dispose cuvette in sharps | □ | □ |
